# Septins tune lipid kinase activity and PI(4,5)P_2_ turnover during G-protein–coupled PLC signalling in vivo

**DOI:** 10.26508/lsa.202101293

**Published:** 2022-03-11

**Authors:** Aastha Kumari, Avishek Ghosh, Sourav Kolay, Padinjat Raghu

**Affiliations:** 1 National Centre for Biological Sciences, TIFR-GKVK Campus, Bengaluru, India; 2 UT Southwestern Medical Center, Dallas, TX, USA; 3 Department of Surgery, Vascular Biology Program, Boston Children’s Hospital, Harvard Medical School, Boston, MA, USA

## Abstract

Phosphatidylinositol 4,5-bisphosphate [PI(4,5)P_2_] hydrolysis by phospholipase C (PLC) is a conserved mechanism of signalling. Given the low abundance of PI(4,5)P_2_, its hydrolysis needs to be coupled to resynthesis to ensure continued PLC activity; however, the mechanism by which depletion is coupled to resynthesis remains unknown. PI(4,5)P_2_ synthesis is catalyzed by the phosphorylation of phosphatidylinositol 4 phosphate (PI4P) by phosphatidylinositol 4 phosphate 5 kinase (PIP5K). In *Drosophila* photoreceptors, photon absorption is transduced into PLC activity and during this process, PI(4,5)P_2_ is resynthesized by a PIP5K. However, the mechanism by which PIP5K activity is coupled to PI(4,5)P_2_ hydrolysis is unknown. In this study, we identify a unique isoform dPIP5K^L^, that is both necessary and sufficient to mediate PI(4,5)P_2_ synthesis during phototransduction. Depletion of PNUT, a non-redundant subunit of the septin family, enhances dPIP5K^L^ activity in vitro and PI(4,5)P_2_ resynthesis in vivo; co-depletion of dPIP5K^L^ reverses the enhanced rate of PI(4,5)P_2_ resynthesis in vivo. Thus, our work defines a septin-mediated mechanism through which PIP5K activity is coupled to PLC-mediated PI(4,5)P_2_ hydrolysis.

## Abstract (precis)

In *Drosophila* photoreceptors, PI(4,5)P_2_ synthesis during PLC signalling is regulated by PIP5K and septins.

## Introduction

In many eukaryotic cells the binding of extracellular ligands to plasma membrane receptors is transduced by the activation of phospholipase C (PLC). PLC hydrolyzes the membrane lipid phosphatidylinositol 4, 5-bisphosphate [PI(4,5)P_2_] on the inner leaflet of the plasma membrane to generate inositol 1,4, 5 trisphosphate (IP_3_) and diacylglycerol (DAG), both of which encode information leading to cellular responses (Berridge, 2009). PI(4,5)P_2_ is present in low amounts at the plasma membrane and during the high rates of PLC activation seen during cell surface receptor activation, PI(4,5)P_2_ at the plasma membrane would be rapidly depleted leading to loss of signalling sensitivity. However, PI(4,5)P_2_ levels at the plasma membrane are relatively stable and the depletion of this lipid during cell PLC activation is typically only transient. Thus, the utilization of PI(4,5)P_2_ by PLC is coupled to the synthesis of this lipid; in eukaryotes, the principal route of PI(4,5)P_2_ synthesis is through the sequential phosphorylation of phosphatidylinositol (PI) at positions 4 and 5 by phosphatidylinositol 4 kinase (Nakatsu et al, 2012; Balakrishnan et al, 2018) and phosphatidylinositol 4 phosphate 5-kinase (Stephens et al, 1991; Chakrabarti et al, 2015) (reviewed in Kolay et al [2016]). However, for these enzymes to resynthesize PI(4,5)P_2_ in response to its utilization by PLC activity, it is necessary that plasma membrane PI(4,5)P_2_ levels are sensed and any drop in the levels of this lipid be communicated to the enzymatic machinery that regulates its synthesis.

*Drosophila* photoreceptors are an influential model for the analysis of G-protein–coupled PLC signalling (Raghu et al, 2012). In these cells, the absorption of light by rhodopsin in photoreceptors is transduced into ion channel activity through G-protein–coupled PLCβ activation leading to the hydrolysis of PI(4,5)P_2_. As part of their visual ecology, *Drosophila* photoreceptors experience bright light illumination leading to high rates of PLC activation (Fain et al, 2010), yet fly photoreceptors do not undergo rapid inactivation and must therefore have mechanisms to sustain PI(4,5)P_2_ levels during high rates of PLCβ activity experienced during bright light illumination. Previous studies have shown that in photoreceptors, during illumination, PI(4,5)P_2_ resynthesis is regulated by a number of molecules including the phosphatidylinositol transfer protein RDGB (Yadav et al, 2015) and PI4KIIIα that generates PI4P (Balakrishnan et al, 2018; Liu et al, 2018). Photoreceptors also express two genes encoding PIP5K activity that is needed to generate PI(4,5)P_2_ from PI4P, *sktl* (Hassan et al, 1998) and *dPIP5K* (Chakrabarti et al, 2015). Although *dPIP5K* null photoreceptors continue to express *sktl*, they show defects in both the electrical response to light and PI(4,5)P_2_ turnover suggesting that this gene plays an indispensable role in generating a pool of PI(4,5)P_2_ that is required for normal phototransduction (Chakrabarti et al, 2015). However, the mechanism by which dPIP5K activity is regulated during light induced PLC activity in photoreceptors remains unknown.

Septins are a group of evolutionarily conserved, GTP-binding proteins first discovered in yeast but now known to be widely distributed in the animal kingdom (Cao et al, 2009). Septins are part of a multiprotein complex composed of multiple classes of subunits; the complex is assembled at the inner leaflet of the plasma membrane using monomers and each class of monomer is composed of multiple members (Fung et al, 2014). They have been implicated in many processes including cell polarity, mitotic spindle positioning and cytokinesis. At the sub-cellular level, septins have been implicated in cytoskeletal organization (Mostowy & Cossart, 2012) through the formation of polymeric, filamentous structures. They have also been studied in the context of plasma membrane compartmentalization through a barrier function, and a role for septins in regulating store operated calcium influx at the plasma membrane has also been reported (Sharma et al, 2013). In mammals, the septin gene family is expanded to encode up to 13 members (Nishihama et al, 2011) divided into four classes and septin oligomers usually include members from each class. In addition to protein interactions between the monomers, septins have also been shown to interact with many cellular proteins and through this mechanism regulate sub-cellular processes (Neubauer & Zieger, 2017). In organisms such as *Drosophila*, the septin gene family is less diverse; for example, the Sept7 subgroup is encoded by a single gene, *pnut* (Neufeld & Rubin, 1994). Null mutations in *pnut* are homozygous lethal during development; however clonal analysis as well as studies of *pnut*^*XP*^*/+* in *Drosophila* have implicated PNUT function in cytoskeletal regulation of cell shape and development in many tissues (Adam et al, 2000; Menon & Gaestel, 2015) and also in the regulation of store operated calcium influx (Deb et al, 2016). Importantly, septin monomers have been shown to bind PI(4,5)P_2_ (Zhang et al, 1999) through an N-terminal phosphoinositide-binding domain. This PI(4,5)P_2_ binding has been shown to modulate the activity of this class of proteins (Tanaka-Takiguchi et al, 2009; Bertin et al, 2010). Thus, septins are a class of proteins localized to the inner leaflet of the plasma membrane and bind PI(4,5)P_2_. Given that PLC activity also hydrolyzes PI(4,5)P_2_ at the inner leaflet of the plasma membrane, and some septins have been shown to regulate calcium influx downstream of PLC signalling (Sharma et al, 2013), septins are suitable candidates for the regulation of PI(4,5)P_2_ resynthesis. However, to date there is no report of the regulation of PI(4,5)P_2_ levels at the plasma membrane by septins.

In this study, we identify an isoform of dPIP5K, dPIP5K^L^ that is enriched in the eye and is both necessary and sufficient to support PI(4,5)P_2_ synthesis during PLC signalling and a normal response to light. Depletion of PNUT, a member of the septin family, enhances dPIP5K^L^-mediated synthesis of PI(4,5)P_2_ during PLC signalling in photoreceptors. Together, our findings define PNUT as a regulator of PI(4,5)P_2_ resynthesis during G-protein–coupled PLC signalling in vivo.

## Results

### Identification of dPIP5K^L^, a novel eye enriched isoform of dPIP5K

**Figure S1.**
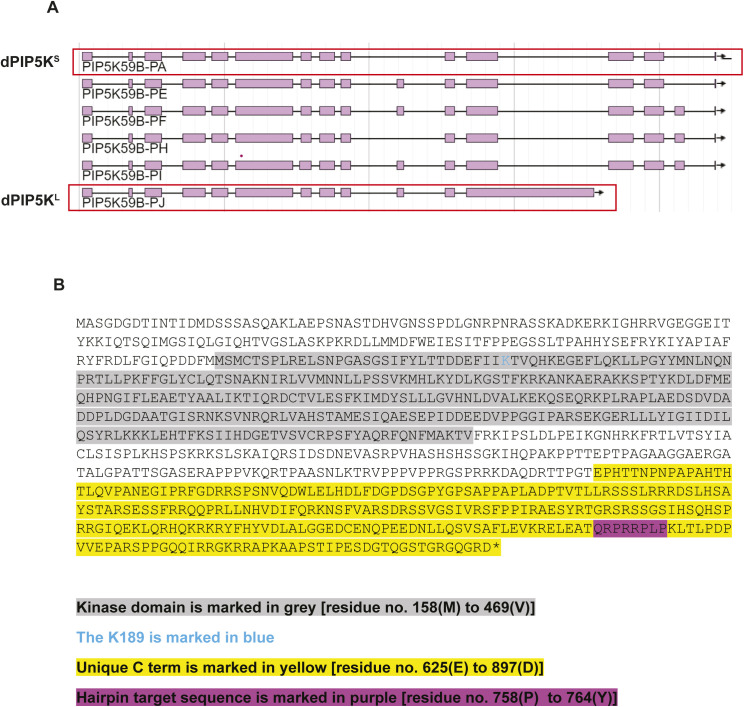
Gene structure and sequence information of *dPIP5K^L^*. **(A)** A schematic adapted from Flybase (version FB2021_06), showing all the predicted splice variants of dPIP5K (PIP5K59B). The splice forms corresponding to dPIP5K^S^(PA) and dPIP5K^L^(PJ) are highlighted. **(B)** Full protein sequence of dPIP5K^L^. The kinase domain is highlighted in grey and the unique C terminal region of dPIP5K^L^ is marked in yellow. The K189, used to make point mutant to generate kinase dead flies is highlighted in blue. Purple marks the residues targeted by hairpin construct against the C terminal of dPIP5K^L^.

**Figure 1.**
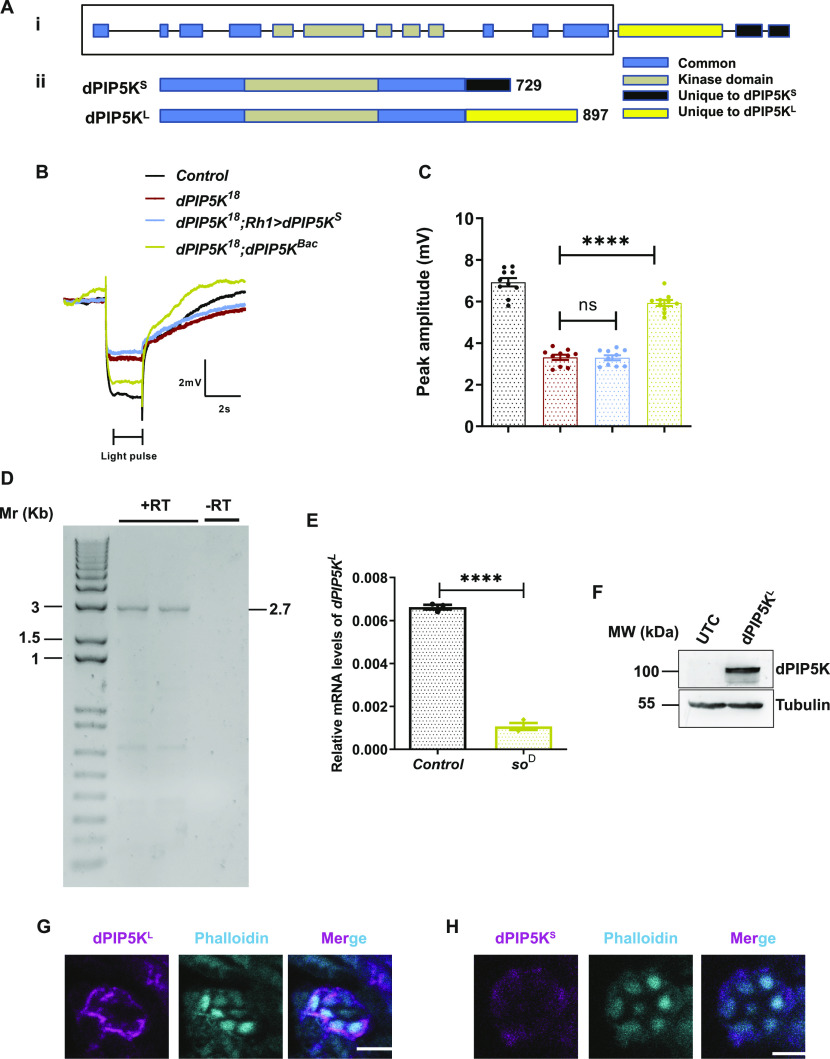
*dPIP5K*^*L*^ is an eye -enriched isoform of *dPIP5K*. **(A)** (i) Schematic illustrating the genomic structure of the *dPIP5K* gene. Exons are represented as rectangles and dashed lines mark the introns. The region common to both isoforms is marked by the black rectangular box. (ii) The protein structure of the two isoforms: dPIP5K^S^ and dPIP5K^L^ is shown (729 and 897 amino acids long, respectively). Domains that are identical between both isoforms are marked in blue, grey marks the common kinase domain, the C terminal region of dPIP5K^S^ is marked in black (132 amino acids), and the C terminal of dPIP5K^L^ is marked in yellow (273 amino acids). **(B)** Representative electroretinogram (ERG) trace from 1-d-old flies of the indicated genotypes. The duration of the stimulus (a 2 s flash of green light) is shown. Y-axis shows response amplitude of the electrical response to light (mV), X-axis indicates duration of the recording(s). In contrast to the *Control*, *dPIP5K*^*18*^ shows a diminished response amplitude. The on/off transients, corresponding to synaptic activity, are also absent in *dPIP5K*^*18*^. Expression of *dPIP5K*^*BAC*^ in *dPIP5K*^*18*^ could rescue the ERG defects whereas expression of *dPIP5K*^*S*^ could not rescue the defects in ERG response. **(C)** Quantification of the peak amplitude for ERG response for the genotypes mentioned in Fig 1B. Y-axis represents peak amplitude and X-axis shows the genotypes. Each data point represents an individual fly tested (n = 10 flies). Error bars represent SEM. **(D)** Agarose gel image showing the amplification of cDNA for *dPIP5K*^*L*^ at 2.7 Kb. +RT and −RT represent presence or absence of the reverse transcriptase enzyme in the reaction mix, respectively. Amplification of the cDNA is seen only in the +RT reactions (loaded in duplicates). **(E)** Quantitative reverse transcription PCR from RNA extracted from the heads of 1-d-old flies showing enrichment of *dPIP5K*^*L*^ transcript in wild-type heads over *so*^*D*^. Y-axis represents relative transcript level for *dPIP5K*^*L*^, normalized to internal control (RP49: ribosomal protein 49). X-axis represents the genotypes (n = 3 biological replicates). Error bars represent SEM. **(F)** Western blot from S2R+ cell extract prepared after transfecting cells with *dPIP5K*^*L*^, probed with anti-dPIP5K antibody (Chakrabarti et al, 2015). A 100-kD band corresponding to dPIP5K^L^ is observed in transfected cells. Tubulin at 55 kD is used as loading control. UTC-untransfected control. **(G, H)** Confocal images of retinae from 1-d-old transgenic flies overexpressing dPIP5K^L^::GFP (G) or dPIP5K^S^::HA (H) in *dPIP5K*^*18*^ background. Phalloidin (cyan) marks the location of the rhabdomere in each photoreceptor. dPIP5K^L^ is detected using anti GFP antibody (magenta) and dPIP5K^S^ is detected using anti HA antibody (magenta). Scale bar = 5 μm (for G and H). Data information: XY plots and scatter dot plots with mean ± SEM are shown. Statistical tests: (E) two-tailed unpaired *t* test with Welch correction. **(C)** One-way ANOVA with post hoc Tukey’s multiple pairwise comparisons. ns, Not significant; ****P < 0.0001.

dPIP5K is an eye enriched PIP5K which produces PI(4,5)P_2_ required for normal phototransduction (Chakrabarti et al, 2015). In a loss of function mutant of *dPIP5K* (*dPIP5K*^*18*^) the electrical response to light is severely reduced. *dPIP5K* has multiple predicted splice variants (Flybase-version FB2021_06: http://fb2021_06.flybase.org/) (Figs S1A and 1A). We observed that the reduced light response could be rescued by re-expressing all the isoforms of dPIP5K using a bacterial artificial chromosome rescue construct (Venken et al, 2006) for dPIP5K (*dPIP5K*^*BAC*^, see the Materials and Methods section for fly details). However, re-expression of a single isoform (*dPIP5K*^*S*^) was unable to restore the light response (Fig 1B and C). Based on the differential rescue and given that the *dPIP5K* gene is alternatively spliced, it seemed possible that a splice variant other than *dPIP5K*^*S*^ must be required to support PI(4,5)P_2_ resynthesis during phototransduction. To identify such an isoform, we reverse transcribed total RNA extracted from 1-d old adult fly retinae using gene specific primers, designed based on the genomic sequence of the *dPIP5K* locus. Sequence analysis of the amplified cDNA product revealed a novel isoform (Full sequence in Fig S1B), distinct from the already known isoform *dPIP5K*^*S*^. This transcript encoded a unique C-terminus of 273 amino acids previously not reported for existing isoforms. Therefore, we named this longer isoform as *dPIP5K*^*L*^ and the known shorter isoform as *dPIP5K*^*S*^ (Figs 1A and D and S1). Through a comparison of RNA extracted from wild-type and *so*^*D*^ mutant (without eyes) heads, we also found that the *dPIP5K*^*L*^ transcript is enriched in the eye (Fig 1E). Protein blots from *Drosophila* S2R+ cells transfected with *dPIP5K*^*L*^ show the expression of a protein with the predicted molecular mass of 100 kD (Fig 1F). Previously we have reported that dPIP5K is localized to the microvillar plasma membrane, using an anti-dPIP5K antibody (Chakrabarti et al, 2015). That antibody was raised against a common region of both dPIP5K^L^ and dPIP5K^S^ and hence could not be used to detect specific isoforms by immunofluorescence. To detect the localization of each isoform, we generated epitope-tagged constructs for each of them (dPIP5K^S^::HA and dPIP5K^L^::GFP). When reconstituted individually into *dPIP5K*^*18*^ (a protein null allele) photoreceptors, we found that dPIP5K^L^ localizes to the plasma membrane at the base of the microvilli (Fig 1G), whereas dPIP5K^S^ was distributed throughout the cell body of photoreceptors, away from the microvillar membrane (Fig 1H). These observations prompted us to study if dPIP5K^L^, with its unique C terminal domain, could be relevant to phototransduction.

### *dPIP5K*^*L*^ encodes a polypeptide with PIP5K activity in vitro

**Figure 2.**
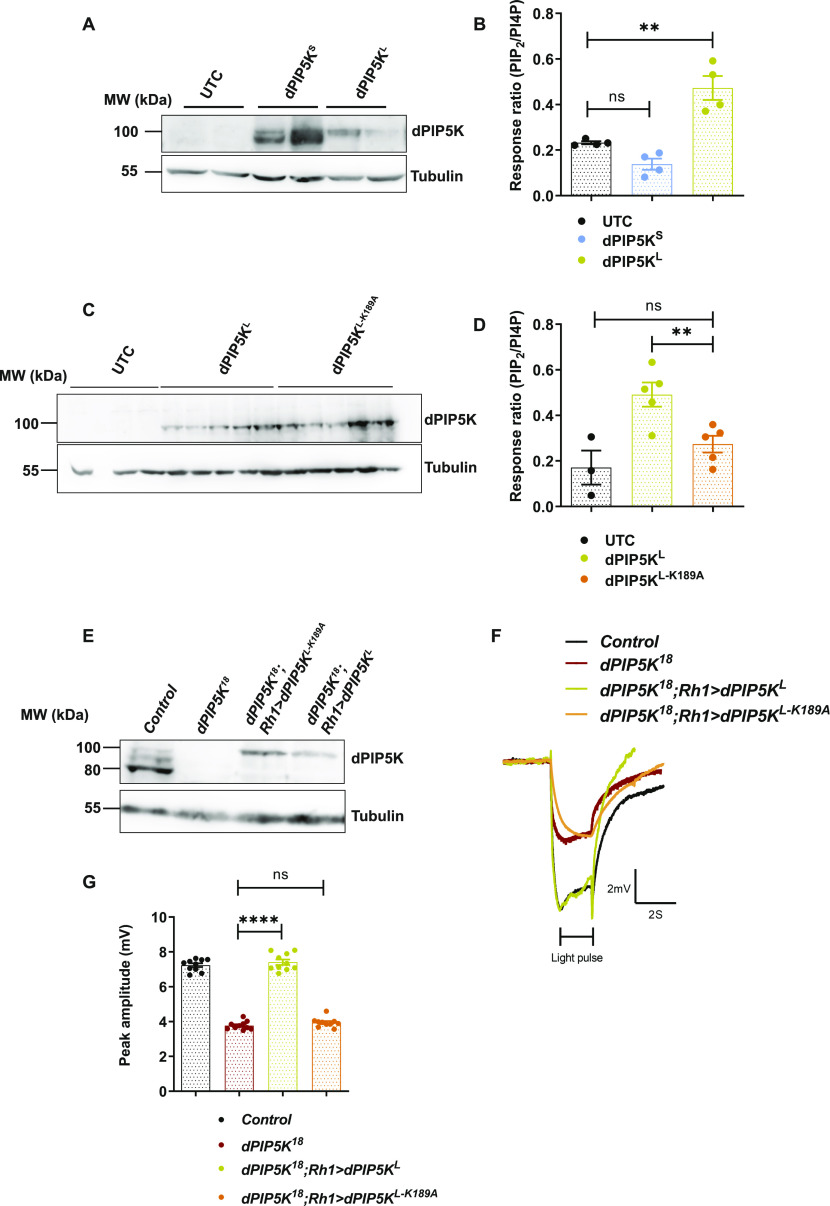
dPIP5K^L^ shows PIP5K activity in vitro and is sufficient to sustain phototransduction in vivo. **(A)** Representative Western blot showing expression of dPIP5K^L^ and dPIP5K^S^ from S2R+ cells extract transfected with the respective constructs. Samples are loaded in duplicates and probed with dPIP5K antibody. Tubulin at 55 kD is used as loading control. UTC-untransfected control. **(B)** Response ratio of product:substrate [37:4-PI(4,5)P_2_:37:4-PI4P] in an in vitro kinase assay with cell free lysate prepared from S2R+ cells transfected with *dPIP5K*^*L*^ or *dPIP5K*^*S*^ (n = 4 biological replicates). This graph is plotted by pooling data from two experiments. Error bars represent SEM. **(C)** Representative Western blot showing expression of dPIP5K^L^ and dPIP5K^L-K189A^ from S2R+ cells extract transfected with the respective constructs (n = 3 biological replicates for UTC and n = 5 for dPIP5K^L^ and dPIP5K^L-K189A^).The blot is probed with dPIP5K antibody and tubulin at 55 kD is used as loading control. UTC-untransfected control. **(D)** Response ratio of product:substrate [37:4-PI(4,5)P_2_:37:4-PI4P] in an in vitro kinase assay with cell free lysate prepared from S2R+ cells transfected with *dPIP5K*^*L*^ or *dPIP5K*^*L-K189A*^ (n = 3 biological replicates for UTC and n = 5 for dPIP5K^L^ and dPIP5K^L-K189A^). Error bars represent SEM. **(E)** Western blot from 1-d old fly retinae showing expression of dPIP5K^L^ and dPIP5K^L-K189A^ in *dPIP5K*^*18*^ background (100 kD band). In *Control*, the band corresponding to dPIP5K^S^ is seen at 80 kD. Tubulin at 55 kD is used as loading control. **(F)** Representative ERG trace from 1-d old flies of the indicated genotypes. The duration of the stimulus (a 2 s flash of green light) is shown. Y-axis shows ERG amplitude (mV), X-axis shows duration of the recording (s). **(G)** Quantification of the peak amplitude for ERG response in each of the mentioned genotypes. Y-axis represents peak amplitude and X-axis shows the genotypes. Each data point represents an individual fly tested (n = 10 flies). Error bars represent SEM. Data information: XY plots and scatter dot plots with mean ± SEM are shown. Statistical tests: (B, D, G) One-way ANOVA with post hoc Tukey’s multiple pairwise comparisons. ns, Not significant; **P < 0.01; ****P < 0.0001.

To understand the functions of dPIP5K^L^ and dPIP5K^S^, we measured their enzymatic activities. PIP5K enzymes use PI4P as a substrate and generate PI(4,5)P_2_. *Drosophila* S2R+ cells do not express dPIP5K. For the enzymatic assay, we used the cell lysate from *Drosophila* S2R+ cells transiently expressing either *dPIP5K*^*L*^ or *dPIP5K*^*S*^ (Fig 2A). Using these lysates, we set up an in vitro kinase assay to quantify the extent of PI(4,5)P_2_ produced by the conversion of the exogenously supplied substrate 37:4 PI4P to PI(4,5)P_2_. The synthetic substrate and product of unique molecular mass was detected and quantified by LC–MS/MS. The extent of enzymatic conversion was measured by the response ratio which is derived by dividing the area under the curve (AUC) of synthetic PI(4,5)P_2_ signal by the AUC of synthetic PI4P signal. Under these in vitro assay conditions, we observed that the response ratio of *dPIP5K*^*L*^ transfected lysates was significantly higher than that from untransfected cell lysates, signifying a high PIP5K activity (Fig 2B). Also, dPIP5K^S^ was catalytically inactive in vitro; the response ratio was not different between *dPIP5K*^*S*^ transfected cell lysates and those from untransfected controls (Fig 2B). The baseline PIP5K activity seen in untransfected cell lysates most likely arises from the endogenous PIP5K expressed in these cells encoded by *sktl* (Hassan et al, 1998), the only other gene in the *Drosophila* genome that encodes a PIP5K enzyme. We also confirmed that the PIP5K activity of dPIP5K^L^ is due to its kinase domain as a catalytically dead version of dPIP5K^L^ (dPIP5K^L-K189A^) did not elicit a significantly different response ratio compared with untransfected controls (Fig 2C and D). Thus, we conclude that dPIP5K^L^ has a high PIP5K activity in vitro whereas dPIP5K^S^ by itself does not exhibit PIP5K activity.

### dPIP5K^L^ is sufficient to sustain phototransduction

**Figure S2.**
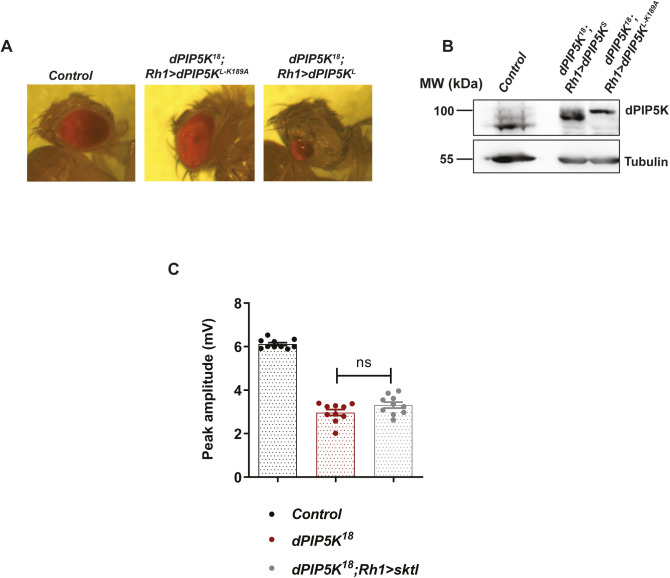
Morphological defects observed in the adult eye upon expression of dPIP5K^L^ in *dPIP5K^18^* at 25^0^C. **(A)** Images showing eye morphology of freshly eclosed flies of the mentioned genotypes. The full-sized red eye of control flies is shown; eye with morphological defect is seen on overexpressing dPIP5K^L^ but not upon overexpression of dPIP5K^L-K189A^. **(B)** Western blot from retinal extracts of 1-d-old fly of the mentioned genotypes showing expression of either dPIP5K^S^::HA (84 kD) or dPIP5K^L-K189A^ (100 kD) in *dPIP5K*^*18*^ background. Tubulin at 55 kD is used as loading control. **(C)** Quantification of the peak amplitude for ERG response in each of the mentioned genotypes. Y-axis represents peak amplitude and X-axis shows the genotypes. Each data point represents an individual fly tested. (n = 10 flies). Error bars represent SEM. Data information: Scatter dot plot with mean ± SEM is shown. Statistical tests: (C) One-way ANOVA with post hoc Tukey’s multiple pairwise comparisons. ns, Not significant.

To study the functional relevance of dPIP5K^L^, we tested if it could rescue the defects in the electrical response to light in the eye (measured using an electroretinogram-ERG) seen in *dPIP5K*^*18*^. We expressed dPIP5K^L^ in *dPIP5K*^*18*^. We observed that most of the flies eclosed with eyes having morphological defects, hence could not be used for ERG recordings; this phenotype is likely due to altered developmental events and was dependent on the kinase activity of dPIP5K^L^ as it was not seen on expression of dPIP5K^L-K189A^ (Fig S2A). However, on rearing the cross at 18°C during development, we could obtain some escapers with normal eye morphology. Western blot analysis of lysates from the eyes of such animals showed the selective re-expression of the dPIP5K^L^ isoform (Fig 2E). ERG recordings from these flies showed that expressing dPIP5K^L^ alone in *dPIP5K*^*18*^ flies completely rescues both the amplitude as well as transients in the ERG (Fig 2F and G). We also observed that when expressed in *dPIP5K*^*18*^, dPIP5K^L-K189A^, which is catalytically inactive (Figs 2E and S2B) was unable to rescue the ERG phenotype (Fig 2F and G). We found that SKTL, the other PIP5K, failed to rescue the ERG phenotype (Fig S2C). These findings demonstrate that dPIP5K^L^ is sufficient to sustain a normal electrical response to light, a function that is dependent on its kinase activity.

### dPIP5K^L^ is essential for normal phototransduction

**Figure S3.**
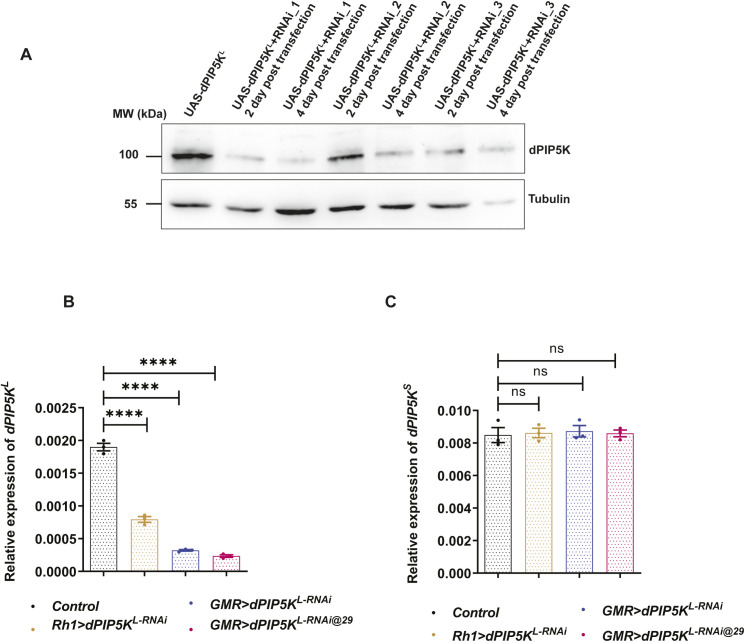
Characterization of dPIP5K^L^ hairpin constructs. **(A)** Western blot from S2R+ cell extract upon co-transfection with *dPIP5K*^*L*^ and hairpin constructs against the C terminal region of dPIP5K^L^ is shown. dPIP5K^L^ is detected at 100 kD and Tubulin at 55 kD is used as loading control. Construct 1 (RNAi_1) gives the maximum knockdown. **(B)** Quantitative real-time PCR from retinae of 1-d-old flies showing the degree of *dPIP5K*^*L*^ transcript knockdown for the mentioned genotypes. Y-axis represents relative transcript level for *dPIP5K*^*L*^, normalized to internal control (*RP49*: ribosomal protein 49). X-axis represents the genotypes (n = 3 biological replicates). Error bars represent SEM. **(C)** Quantitative real-time PCR from retinae of 1-d-old flies showing no change of transcript level of *dPIP5K*^*S*^ upon knockdown of dPIP5K^L^ in the mentioned genotypes. Y-axis represents relative transcript level for *dPIP5K*^*S*^, normalized to internal control (*RP49*: ribosomal protein 49). X-axis represents the genotypes (n = 3 biological replicates). Error bars represent SEM. Data information: Scatter dot plots with mean ± SEM are shown. Statistical tests: (B, C) One-way ANOVA with post hoc Tukey’s multiple pairwise comparisons. ns, Not significant; ****P < 0.0001.

**Figure 3.**
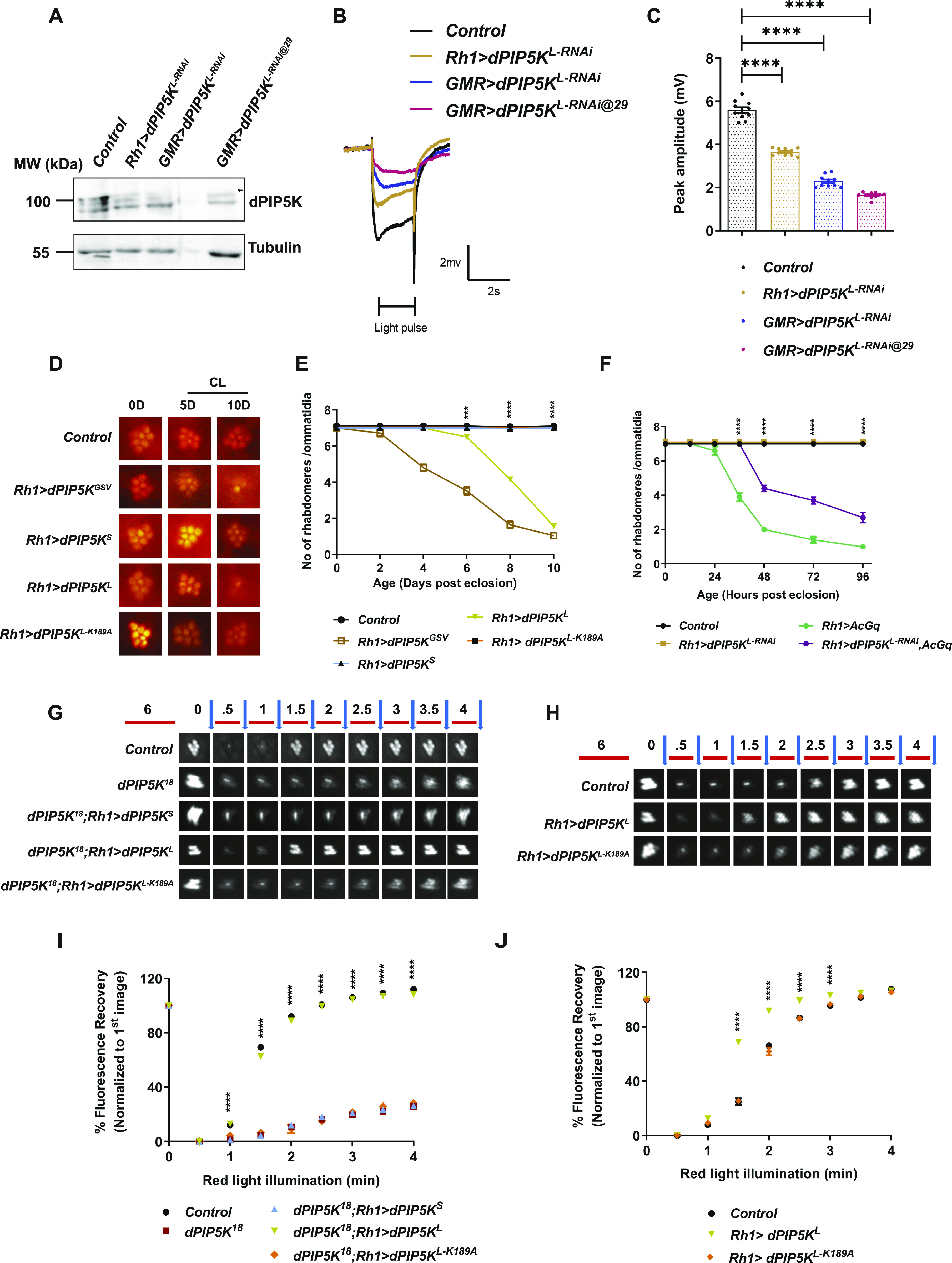
The kinase activity of dPIP5K^L^ is essential for phototransduction. **(A)** Western blot from 1-d old fly retinae showing the degree of knockdown of dPIP5K^L^ (100 kD, topmost band, marked by arrowhead) in the mentioned genotypes upon expression of an RNAi to this isoform; blot was probed with dPIP5K antibody. Tubulin at 55 kD is used as loading control. All the crosses for RNAi were reared in 25° incubators except for GMR>dPIP5K^L-RNAi@29^, which was reared at 29°C, to enhance the efficacy of the RNAi. **(B)** Representative ERG trace from 1-d-old flies of the indicated genotypes. The duration of the stimulus (a 2 s flash of green light) is shown. Y-axis shows response amplitude in mV, X-axis shows duration of the recording (s). **(C)** Quantification of the peak amplitude for ERG response in each of the mentioned genotypes. Y-axis represents peak amplitude and X-axis shows the genotypes. Each data point represents an individual fly tested (n = 10 flies). Error bars represent SEM. **(D)** Representative optical neutralization images for the mentioned genotypes. Flies were grown in constant light at 2,000 lux; age in days (D) is mentioned at the top of each panel. 0D is the day of eclosion, 5D-5 days, and 10D-10 days post eclosion, CL-constant light. **(E, F)** Graphs showing quantification for the time course of retinal degeneration on overexpression of the mentioned transgenes under constant light conditions. (light intensity: [E] 2000 lux; [F] 5,500 lux). 10 ommatidia from 5 separate flies of each genotype were scored and plotted. Y-axis is the number of intact rhabdomeres/ommatidium. The maximum value possible is 7. X-axis is the age of the flies post-eclosion. Error bars represent SEM. **(G, H)** Deep pseudopupil imaging of PI(4,5)P_2_ levels in the microvillar membrane of photoreceptors. The fluorescence of the PH-PLCδ::GFP probe is depicted. The protocol used is represented: Red light illumination periods are shown as red bars, with the numbers on top representing illumination time in minutes. Flashes of blue light (blue arrows) used for image capture are also depicted. Representative fluorescent image captured for each time point for the mentioned genotypes is shown. **(I, J)** Graphs showing quantification of recovery kinetics of the fluorescent deep pseudopupil with time in flies expressing the PI(4,5)P_2_ reporter PH-PLCδ::GFP. Y-axis represents the mean fluorescence intensity of the pseudopupil image at each time point expressed as percentage of the intensity of first image acquired, X-axis represents the red light illumination period in minutes (n = 10 flies). Error bars represent SEM. Data information: XY plots and scatter dot plots with mean ± SEM are shown. Statistical tests: (C) One-way ANOVA with post hoc Tukey’s multiple pairwise comparisons. **(E, F, I, J)** Two-way ANOVA grouped analysis with Bonferroni’s post multiple-comparisons tests to compare means. ***P < 0.001; ****P < 0.0001. The statistical significance for the following data pairs is represented on the graphs: (E) *Control* versus *Rh1>dPIP5K*^*L*^; (F) *Rh1>Ac*_*Gq*_ versus *Rh1>dPIP5K*^*L*^, *Ac*_*Gq*_; (I) *dPIP5K*^*18*^ versus *dPIP5K*^*18*^*;Rh1>dPIP5K*^*L*^; (J) *Control* versus *Rh1>dPIP5K*^*L*^.

Next, we checked whether dPIP5K^L^ is essential for phototransduction. To answer this question, we designed short hairpin constructs that would specifically target and knockdown dPIP5K^L^ by targeting the exon coding for the unique C terminus region of dPIP5K^L^ (Ni et al, 2009; Perkins et al, 2015). To test the effectiveness of these hairpin constructs, a Western blot was performed from *Drosophila* S2R+ cells co-transfected with *dPIP5K*^*L*^ and each of the hairpin constructs to check for dPIP5K^L^ protein knockdown. Of the three hairpins we designed, construct 1 showed the highest level of knockdown (Fig S3A) and hence we generated transgenic fly lines with it. We used two different eye specific enhancers that differ in terms of strength and timing of expression (GMR-expression starts third larval instar onwards and Rh1 expression starts 70% pupal development onwards) to deplete *dPIP5K*^*L*^ transcript (Fig S3B) and this protein isoform was also depleted (Fig 3A). The transcript level of *dPIP5K*^*S*^ was not affected (Fig S3C). We observed that with increasing degree of dPIP5K^L^ depletion, the amplitude of the ERG response diminishes (Fig 3B and C). Furthermore, upon knockdown with *Rh1* enhancer, which starts expressing from 70% pupal development, about 70% of the eclosed flies showed a lower ERG amplitude when compared with controls but with normal on/off transients (that report normal synaptic transmission), implying that the diminished amplitude upon depletion of dPIP5K^L^ is not a downstream consequence of altered synaptic transmission but rather the protein is required to sustain a normal receptor potential during the light response. These experiments demonstrate that dPIP5K^L^ is both necessary and sufficient to sustain a normal electrical response to light.

Light-dependent retinal degeneration, or collapse of the apical photoreceptor membrane post development in adult flies, is a phenotype frequently associated with changes in gene products that are a part of phototransduction (reviewed in Raghu et al [2012]). When all isoforms of dPIP5K (*Rh1>dPIP5K*^*GSV*^) are overexpressed in photoreceptors using *dPIP5K*^*GSV*^ (Toba et al, 1999), a strong light dependent retinal degeneration is seen (Fig 3D and E). To test the contribution of each isoform to this phenotype, in this study, we overexpressed them individually and found that overexpression of dPIP5K^L^ (*Rh1>dPIP5K*^*L*^) led to light dependent retinal degeneration (Fig 3D and E), whereas overexpression of dPIP5K^S^ did not result in this phenotype (*Rh1>dPIP5K*^*S*^) (Fig 3D and E). The ability of dPIP5K^L^ overexpression to trigger retinal degeneration was dependent on kinase activity of the enzyme because overexpression of dPIP5K^L-K189A^, a kinase dead version, did not show any degeneration (Fig 3D and E).

The heterotrimeric G-protein α subunit, Gq, is essential for PLCβ activity which in turn is required for phototransduction (Scott et al, 1995). Expression of a constitutively active form of Gq (Ac_Gq_) hyperstimulates phospholipase Cβ leading to excessive electrical activity and triggering retinal degeneration (Lee et al, 1994). If dPIP5K^L^ generates the PI(4,5)P_2_ pool required for PLCβ activity, then one might predict that depletion of this isoform, thus reducing the availability of PI(4,5)P_2_ required for PLCβ activity, might suppress the effects of Ac_Gq_ expression. Consistent with this, when Ac_Gq_ (Ratnaparkhi et al, 2002) was expressed in photoreceptors (*Rh1>Ac*_*Gq*_), we found that when reared in light, these photoreceptors underwent retinal degeneration (Fig 3F), and this retinal degeneration was slowed when dPIP5K^L^ was depleted in photoreceptors (Fig 3F).

### dPIP5K^L^ is necessary and sufficient to support PI(4,5)P_2_ turnover during phototransduction

dPIP5K^L^ and dPIP5K^S^ showed a differential ability to rescue the electrical response to light, a process that depends critically on normal PI(4,5)P_2_ turnover during phototransduction. It has previously been reported that in the loss-of-function mutant of *dPIP5K* (*dPIP5K*^*18*^), the kinetics of PI(4,5)P_2_ turnover is severely impaired (Chakrabarti et al, 2015). To test if this differential rescue of the ERG amplitude by dPIP5K^L^ and dPIP5K^S^ correlated with their ability to support PI(4,5)P_2_ turnover, we measured PI(4,5)P_2_ levels at the microvillar membrane in vivo, using a PI(4,5)P_2_ reporter, PH-PLCδ::GFP, whose fluorescence intensity is a read out for (PI4,5P)_2_ levels at the microvillar plasma membrane (see the Materials and Methods section for details). When *dPIP5K*^*L*^ was selectively expressed in *dPIP5K*^*18*^, the delayed kinetics of PI(4,5)P_2_ re-synthesis in this mutant was rescued; by contrast selective expression of *dPIP5K*^*L-K189A*^ or *dPIP5K*^*S*^ failed to do so (Fig 3G and I). Moreover, upon overexpression of dPIP5K^L^ in otherwise wild-type flies, the kinetics of PI(4,5)P_2_ re-synthesis was accelerated; this was dependent on the kinase activity of dPIP5K^L^ because overexpression of dPIP5K^L-K189A^ (*Rh1> dPIP5K*^*L-K189A*^) did not accelerate the resynthesis of PI(4,5)P_2_ levels (Fig 3H and J). These observations indicate that dPIP5K^L^ is both necessary and sufficient to control PI(4,5)P_2_ resynthesis after light-induced PLCβ activity.

### Septin 7 depletion alters the light response and PI(4,5)P_2_ turnover

**Figure 4.**
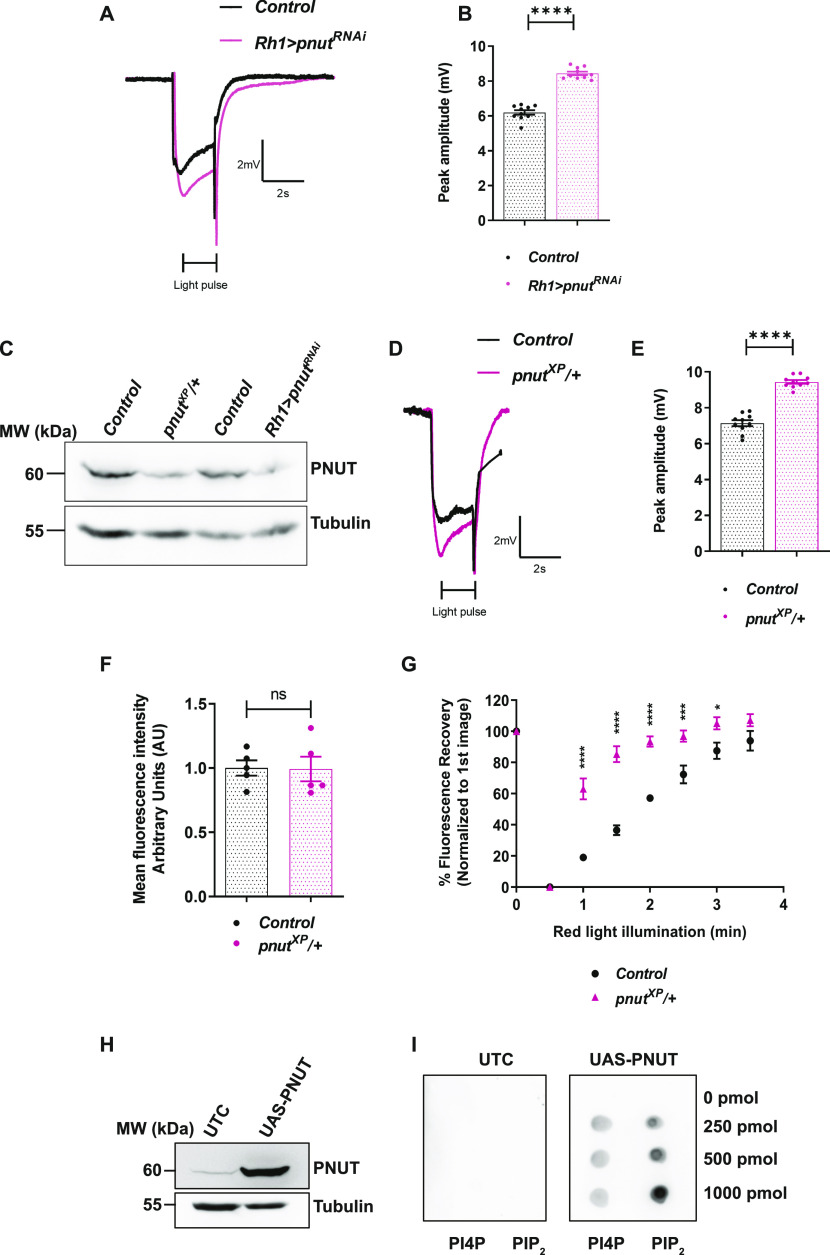
PNUT depletion alters light response and PI(4,5)P_2_ turnover. **(A, D)** Representative ERG trace from 1-d old flies of the indicated genotypes. Both *pnut*^*XP*^*/+* and *Rh1>pnut*^*RNAi*^ show enhanced amplitude response w.r.t *Control*. The 2 s stimulus with a flash of green light is shown. Y-axis shows response amplitude (mV), X-axis indicates duration of the recording (s). **(B, E)** Quantification of the peak amplitude for ERG response in each of the mentioned genotypes. Y-axis represents peak amplitude and X-axis shows the genotypes. Each data point represents an individual fly tested (n = 10 flies). Error bars represent SEM. **(C)** Western blot from retinal extracts of 1-d old flies of the mentioned genotypes. Anti-PNUT detects a band at 60 kD. Tubulin at 55 kD is used as loading control. **(F)** Quantification of the mean fluorescence intensity of the deep pseudopupil image in flies expressing the PI(4,5)P_2_ reporter PH-PLCδ::GFP. Y-axis represents mean fluorescence intensity represented in arbitrary units, X-axis shows the genotypes (n = 5). Each intensity value has been normalized to the mean of *Control*. Error bars represent SEM. **(G)** Graph showing quantification of recovery kinetics of fluorescent pseudopupil with time in flies expressing the PI(4,5)P_2_ reporter PH-PLCδ::GFP. Y-axis represents the mean fluorescence intensity of the pseudopupil expressed as percentage of the intensity of first image acquired, X-axis represents the red-light illumination period in minutes (n = 5 flies). Error bars represent SEM. **(H)** Representative Western blot showing expression of PNUT from S2R+ cells extract, either untransfected (UTC) or transfected with *UAS-PNUT* construct. Anti-PNUT detects a band at 60 kD. Tubulin at 55 kD is used as loading control. **(I)** Nitrocellulose membrane spotted with increasing concentrations of PI4P and PI(4,5)P_2_, incubated with either untransfected S2R+ cells extract (UTC) or cells extract expressing PNUT. Binding was detected using the anti PNUT antibody. This experiment was repeated twice. Data information: XY plots and scatter dot plots with mean ± SEM are shown. Statistical tests: (B, E, F) two-tailed unpaired *t* test with Welch correction. **(G)** Two-way ANOVA grouped analysis with Bonferroni’s post multiple-comparisons tests to compare means. ns, Not significant; *P < 0.05; ***P < 0.001; ****P < 0.0001.

**Figure S4.**
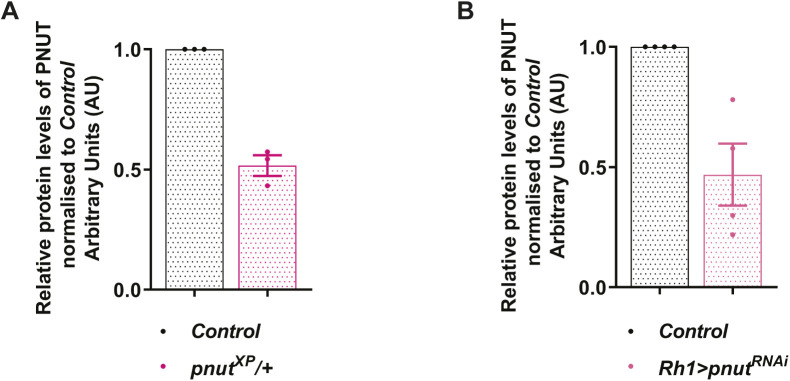
Densitometry based quantification of PNUT levels. **(A, B)** Graphs showing densitometric quantification of relative protein levels of PNUT in the mentioned genotypes normalized to *Control*. Y-axis represents relative protein levels in arbitrary units (AU), X-axis shows the genotypes. The graphs here represent quantification from three independent Western blots for (A), and four independent Western blots for (B). Error bars represent SEM. Data information: Scatter dot plots with mean ± SEM are shown.

A previous study has implicated septin function in the regulation of PI(4,5)P_2_ turnover during G-protein–coupled PLCβ signalling at the plasma membrane (Sharma et al, 2013). To examine if septins have any role in regulating PI(4,5)P_2_ turnover during phototransduction, we performed an RNAi depletion based screen against all *Drosophila* septins looking for an effect on the amplitude of the electrical response to light. From this study, we found that knockdown of the sole class 7 septin in *Drosophila* (*pnut*) (Figs 4C and S4B) led to an enhanced ERG amplitude (Fig 4A and B). Null mutants of *pnut* (*pnut*^*XP*^) have previously been generated; homozygous *pnut*^*XP*^ mutants are not viable as adult flies and homozygous mosaic eyes of this allele impact eye development (Neufeld & Rubin, 1994) and preclude analysis of signalling in homozygous adult photoreceptors. However, in Western blots from *Drosophila* heads, *pnut*^*XP*^/+ flies show circa 50% of the protein levels as compared with wild-type controls (Figs 4C and S4A) and photoreceptors are morphologically normal. We found that on stimulation with equal intensities of light, *pnut*^*XP*^/+ flies have an enhanced ERG amplitude compared with controls (Fig 4D and E). Thus, depletion of PNUT results in an altered electrical response to light. We also measured PI(4,5)P_2_ turnover in PNUT-depleted photoreceptors; resting levels of PI(4,5)P_2_ were no different between *pnut*^*XP*^/+ and controls (Fig 4F); however, after PLCβ activation by illumination, PI(4,5)P_2_ levels recovered much faster in *pnut*^*XP*^/+ compared with controls (Fig 4G).

Septin monomers have been shown to bind PI(4,5)P_2_ (Zhang et al, 1999) and this binding can modulate the activity of this class of proteins (Tanaka-Takiguchi et al, 2009; Bertin et al, 2010) raising the attractive possibility that they may form the molecular link between PLCβ activity and PI(4,5)P_2_ levels at the plasma membrane. We tested if the PI(4,5)P_2_ binding activity reported for yeast septins is conserved in PNUT. We incubated nitrocellulose membrane spotted with increasing concentrations of PI(4,5)P_2_ with extracts of S2R+ cells overexpressing PNUT (Fig 4H). We found that PNUT shows a concentration-dependent binding to PI(4,5)P_2_ (Fig 4I). Thus, PNUT can bind PI(4,5)P_2_ and depletion of PNUT enhances PI(4,5)P_2_ turnover during PLCβ signalling in photoreceptors.

### PNUT regulates PI(4,5)P_2_ synthesis via dPIP5K^L^ and downstream of RDGB function

**Figure S5.**
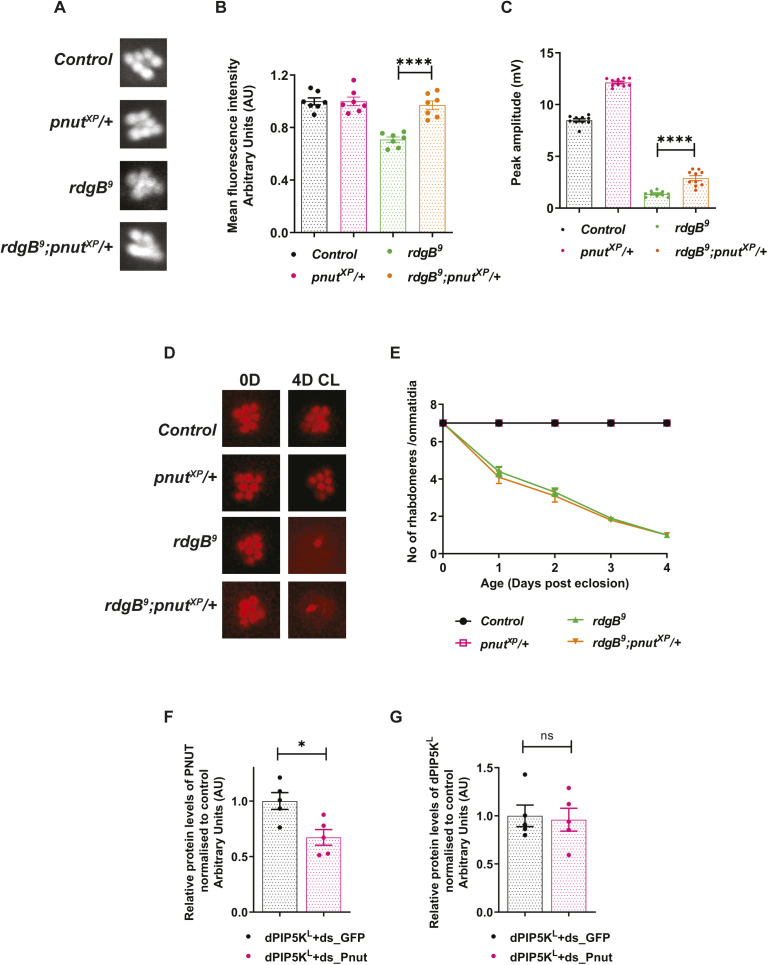
PNUT acts downstream of RDGB. **(A)** Representative images of fluorescent deep pseudopupil from 1 d old flies of the mentioned genotypes expressing the PH-PLCδ::GFP probe. **(B)** Quantification of the mean fluorescence intensity of the deep pseudopupil image in flies expressing the PI(4,5)P_2_ reporter PH-PLCδ::GFP. Y-axis represents mean fluorescence intensity represented in arbitrary units, X-axis shows the genotypes (n = 7 flies). Each intensity value has been normalized to the mean of *Control*. Error bars represent SEM. **(C)** Quantification of the peak amplitude for ERG response in each of the mentioned genotypes. Y-axis represents peak amplitude and X-axis shows the genotypes. Each data point represents an individual fly tested (n = 10 flies). Error bars represent SEM. **(D)** Representative optical neutralization images for the mentioned genotypes. **(D)** Flies were grown in constant light at 2000 lux; age in days (D) is mentioned at the top of each panel. 0D is the day of eclosion, 4D-4 days post eclosion, CL-constant light. **(E)** Graph showing quantification for the time course of retinal degeneration for the mentioned genotypes under constant light conditions. (light intensity: 2,000 lux). The rate of degeneration is similar in *rdgB*^*9*^ and *rdgB*^*9*^*;pnut*^*XP*^*/+* flies. 10 ommatidia from 5 separate flies of each genotype were scored and plotted. Y-axis is the number of intact rhabdomeres/ommatidium. The maximum value possible is 7. X-axis is the age of the flies post-eclosion. Error bars represent SEM. **(F, G)** Graphs showing densitometric quantification of relative protein levels of PNUT and dPIP5K^L^ in the mentioned samples normalized to the mean of *Control* (dPIP5K^L^+ds_GFP). Y-axis represents relative protein levels in arbitrary units (AU), X-axis shows the sample name. **(F)** A significant reduction in PNUT protein levels can be seen in samples treated with dsRNA against PNUT as compared with *Control* samples treated with GFP dsRNA (F). **(G)** However, the protein levels of dPIP5K^L^ remain similar across the samples (G). (n = 5 biological replicates). Error bars represent SEM. Data information: Scatter dot plots and XY plots with mean ± SEM are shown. Statistical tests: (F, G) two-tailed unpaired *t* test with Welch correction. **(B, C)** One-way ANOVA with post-hoc Tukey’s multiple pairwise comparisons. **(E)** Two-way ANOVA grouped analysis with Bonferroni’s post multiple-comparisons tests to compare means. ns, Not significant; *P < 0.05; ****P < 0.0001.

**Figure 5.**
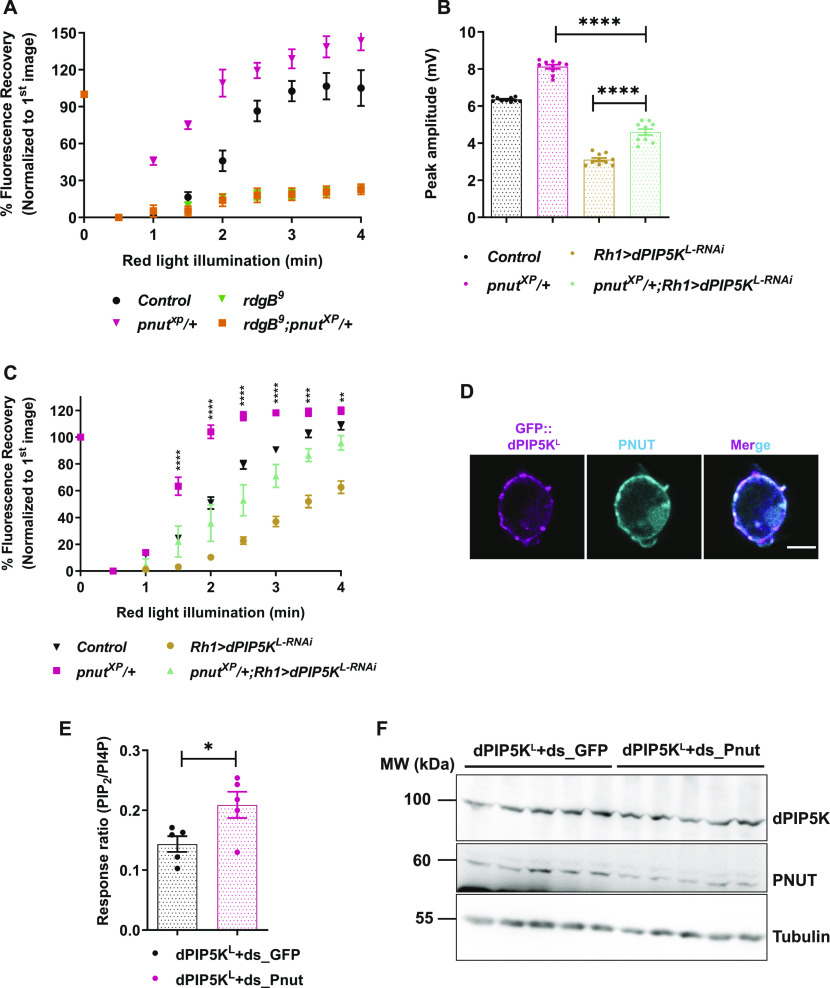
PNUT regulates PI(4,5)P_2_ synthesis via dPIP5K^L^ and downstream of RDGB function. **(A, C)** Graphs quantifying the recovery kinetics of fluorescent pseudopupil with time in flies expressing the PI(4,5)P_2_ reporter PH-PLCδ::GFP. Y-axis represents the mean fluorescence intensity of the pseudopupil image expressed as percentage of the intensity of first image acquired, X-axis represents the red light illumination period in minutes (n = 7 flies for A, 10 flies for C). Error bars represent SEM. **(B)** Quantification of the peak amplitude for ERG response in each of the mentioned genotypes. Y-axis represents peak amplitude and X-axis shows the genotypes. Each data point represents an individual fly tested (n = 10 flies). Error bars represent SEM. **(D)** Representative confocal images of S2R+ cells transfected with *GFP::PIP5K*^*L*^. Localization of endogenous PNUT is detected using the anti-PNUT antibody; GFP::PIP5K^L^ is detected using the anti GFP antibody. Scale bar: 5 μm. **(E)** Response ratio of product:substrate [37:4-PI(4,5)P_2_:37:4-PI4P] in an in vitro kinase assay with cell free lysate prepared from S2R+ cells transfected with *dPIP5K*^*L*^, and treated either with control dsRNA against GFP (dPIP5K^L^+ds_GFP), or with dsRNA against PNUT (dPIP5K^L^+ds_Pnut) (n = 5 biological replicates). This experiment was repeated twice. Error bars represent SEM. **(F)** Western blots showing expression of PNUT from S2R+ cell extract transfected with *dPIP5K*^*L*^, and treated with dsRNA either against PNUT or control GFP. PNUT is detected at 60 kD. Knockdown of PNUT can be seen in ds_PNUT treated cells (dPIP5K^L^+ds_Pnut) as compared with control ds_GFP treated cells (dPIP5K^L^+ds_GFP). dPIP5K^L^ (at 100 kD) is expressed equivalently in both cases. Tubulin at 55 kD is used as loading control. Data information: XY plots and scatter dot plots with mean ± SEM are shown. Statistical tests: (E) two tailed unpaired *t* test with Welch correction. **(B)** One-way ANOVA with post hoc Tukey’s multiple pairwise comparisons. **(A, C)** Two-way ANOVA grouped analysis with Bonferroni’s post multiple-comparisons tests to compare means. *P < 0.05; **P < 0.01; ***P < 0.001; ****P < 0.0001. The statistical significance for the following data pairs is represented on graph (C) *pnut*^*XP*^*/+* versus *pnut*^*XP*^*/+*; *Rh1 dPIP5K*^*L-RNAi*^.

During PLCβ signalling in *Drosophila* photoreceptors, PI(4,5)P_2_ is replenished through the sequential phosphorylation of PI by PI4KIIIα (Balakrishnan et al, 2018) and dPIP5K (Chakrabarti et al, 2015); the PI required as substrate is provided at the plasma membrane by the lipid transfer protein RDGB (Yadav et al, 2015; Raghu et al, 2021). To uncover the step of the phototransduction cycle that could be regulated by PNUT, we tested the effect of PNUT depletion in photoreceptors that were also depleted for RDGB. Previous work has shown that in the strong hypomorph *rdgB*^*9*^, there is a reduction in the electrical response to light, light dependent retinal degeneration and delayed PI(4,5)P_2_ resynthesis (Yadav et al, 2015). In *rdgB*^*9*^*;pnut*^*XP*^*/+*, although the basal PI(4,5)P_2_ levels were similar to controls (Fig S5A and B), the rate of PI(4,5)P_2_ resynthesis and the light dependent retinal degeneration was same as seen in *rdgB*^*9*^ flies (Figs 5A and S5D and E). The ERG amplitude in *rdgB*^*9*^*;pnut*^*XP*^*/+* was only marginally improved compared with *rdgB*^*9*^ alone (Fig S5C). Thus, intact RDGB function is required for the enhanced PI(4,5)P_2_ resynthesis seen in *pnut*^*XP*^*/+*.

Next, we tested if dPIP5K^L^ depletion can impact the consequences of PNUT depletion. When dPIP5K^L^ depletion was performed in photoreceptors that were also *pnut*^*XP*^/+, the enhanced ERG amplitude of *pnut*^*XP*^/+ was suppressed (Fig 5B). To test the relationship of this genetic interaction seen at the level of the ERG amplitude to PI(4,5)P_2_ turnover, we measured PI(4,5)P_2_ turnover kinetics comparing *pnut*^*XP*^*/+* with dPIP5K^L^ knockdown. We found that depletion of dPIP5K^L^ reversed the enhanced PI(4,5)P_2_ recovery kinetics seen in *pnut*^*XP*^*/+* flies (Fig 5C). Thus, the enhanced PI(4,5)P_2_ turnover kinetics seen in *pnut*^*XP*^*/+* requires normal levels of dPIP5K^L^.

To understand the mechanism of the genetic interaction between *pnut* and *dPIP5K*^*L*^ during PLCβ signalling, we studied the localization of these proteins in *Drosophila* S2R^+^ cells; in these cells, endogenous PNUT was found at the plasma membrane and when transfected into these cells, dPIP5K^L^ was also found at this location (Fig 5D). Because our genetic data (Fig 5B and C) suggest a negative interaction between *pnut* and *dPIP5K*^*L*^, we tested if dPIP5K^L^ activity might be altered in PNUT depleted cells. For this, we performed an in vitro kinase assay from *Drosophila* S2R+ cells expressing dPIP5K^L^, where PNUT had been depleted using dsRNA. The degree of dsRNA induced depletion of PNUT protein was quantified (Figs 5F and S5F and G) Under these conditions, the activity of dPIP5K^L^ was significantly increased as compared with control cells (treated with dsRNA against GFP) (Fig 5E).

## Discussion

The activation of PLC is a widespread mechanism of signalling triggered when plasma membrane receptors bind their cognate ligands. Given that the substrate of PLC, PI(4,5)P_2_, is present at the plasma membrane in low amounts, PLC activation can rapidly deplete this lipid leading to loss of signalling function in cells. This situation may arise in settings such as neurons where GPCRs activate PLC at high rates or T-cell receptor activation where sustained activation of PLC occurs over a long period of time. To avoid the depletion of PI(4,5)P_2_, leading to loss of signalling, it is essential that the resynthesis of this lipid is coupled with its consumption by PLC activity. To achieve this, conceptually, cells require a mechanism that couples PLC activation to the enzymatic machinery for synthesis so as to restore PI(4,5)P_2_ levels at the plasma membrane.

In eukaryotic cells, the synthesis of PI(4,5)P_2_ is primarily mediated by the PIP5K class of enzymes (reviewed in Kolay et al [2016]) and in some cells pools of PI(4,5)P_2_ specifically used during PLC signalling are synthesized by PIP5K (Chakrabarti et al, 2015). How is the activity of PIP5K controlled to trigger PI(4,5)P_2_ resynthesis? In this study we report a splice variant of dPIP5K (*dPIP5K*^*L*^), that is both necessary and sufficient to support PI(4,5)P_2_ turnover during *Drosophila* phototransduction, a process in which the plasma membrane of photoreceptors experiences high rates of PLC activation. Although the *dPIP5K* gene is alternately spliced to give two isoforms, dPIP5K^S^ and dPIP5K^L^, only dPIP5K^L^ was enzymatically active in vitro. Consistent with its function during phototransduction, we found that dPIP5K^L^ expression is enriched in *Drosophila* photoreceptors and the protein localizes to the base of the microvillar membrane, close to the sub-cellular site of phototransduction. A number of findings reported here strongly indicate that the kinase activity of dPIP5K^L^ is required for its ability to support PI(4,5)P_2_ synthesis for PLC signalling in vivo: (i) both the reduced electrical response and the delayed kinetics of PI(4,5)P_2_ resynthesis of *dPIP5K* null mutants can be rescued by a wild-type *dPIP5K*^*L*^ transgene but not a point mutant which is kinase dead; (ii) overexpression of wild-type but not kinase-dead dPIP5K^L^ results in light dependent retinal degeneration; and (iii) retinal degeneration triggered by constitutive activation of PLCβ can be rescued by depletion of dPIP5K^L^. By contrast, although it also contains a kinase domain, dPIP5K^S^ did not show kinase activity in vitro and was not able to support PLC signalling in *Drosophila* photoreceptors in vivo. The function of dPIP5K^S^ remains to be determined. However, given that the principal difference between dPIP5K^L^ and dPIP5K^S^ is the C-terminal extension of the longer isoform, it would seem reasonable that this unique C-terminal region of 273 amino acids will be important in the control of enzyme activity and ability to support function in vivo. A conceptually similar observation has been reported in the case of a mammalian PIP5Kγ isoform, where the binding of FERM domain of talin to the C-terminal region of the enzyme has been reported to link this isoform to ongoing integrin signalling (Di Paolo et al, 2002; Ling et al, 2002).

For dPIP5K^L^ to support PI(4,5)P_2_ synthesis, the enzyme needs to be regulated during light induced PLC activation. In a search for molecules that can mediate this regulation, we found that depletion of PNUT, the only septin 7 ortholog in *Drosophila*, results in an enhanced light response and accelerates the resynthesis of PI(4,5)P_2_ at the microvillar plasma membrane after PLC activation. These observations are consistent with a role of PNUT in regulating PI(4,5)P_2_ synthesis after PLC activation. We also found that the enhanced rate of PI(4,5)P_2_ resynthesis seen on PNUT depletion was abolished in *rdgB* mutants. Because the activity of RDGB (phosphatidylinositol transfer protein) supplies PI at the plasma membrane for PI(4,5)P_2_ synthesis (Yadav et al, 2015), this finding suggests that the enhanced PI(4,5)P_2_ synthesis seen in *pnut*^*XP*^*/+* requires ongoing RDGB function and PNUT likely works on a step of PI(4,5)P_2_ resynthesis downstream of RDGB. There are two enzymatic steps downstream of RDGB during the synthesis of PI(4,5)P_2_, the synthesis of PI4P by PI4KIIIα and the phosphorylation of PI4P by PIP5K to generate PI(4,5)P_2_. Our analysis revealed that the accelerated PI(4,5)P_2_ resynthesis seen in *pnut*^*XP*^*/+* could be rescued by depletion of dPIP5K^L^, consistent with the idea that PNUT regulates PI(4,5)P_2_ resynthesis through a mechanism that requires PIP5K activity. We observed that in *Drosophila* S2R+ cells, both PNUT and dPIP5K^L^ are localized to the plasma membrane, consistent with a role for PNUT dependent regulation of PIP5K function. Although PNUT and dPIP5K^L^ co-localized at the plasma membrane, we were not able to demonstrate a direct protein–protein interaction between PNUT and dPIP5K^L^ (data not shown). Thus, the regulation of dPIP5K^L^ by PNUT may be indirect through the function of additional molecules. Many proteins interacting with septins have been described (Neubauer & Zieger, 2017). Indeed in *Drosophila*, PNUT has been shown to interact with septin interacting protein 1 (SIP1) (Shih et al, 2002) and an analysis of the role of such molecules in regulating PLC signalling will likely be useful in understanding regulation of PI(4,5)P_2_ resynthesis by septins.

**Figure 6.**
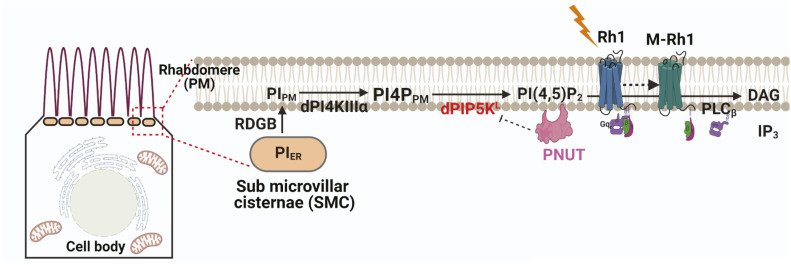
Model for PNUT-mediated regulation of PI(4,5)P_2_ synthesis during G-protein–coupled PLC signalling in *Drosophila* photoreceptors. Towards left, is a cartoon showing cross section of a single *Drosophila* photoreceptor cell with rhabdomere (the apical plasma membrane, PM), sub-microvillar cisternae (SMC) which is a modified smooth ER compartment and cell body. The area indicated by the red box is expanded to show the biochemical reactions involved in the resynthesis of PI(4,5)P_2_. Protein and lipid components are depicted according to the compartment in which they are distributed. During phototransduction, upon light stimulation, activated PLC (PLC_β_) hydrolyses the PI(4,5)P_2_ produced by dPIP5K^L^, depleting the PI(4,5)P_2_ at the plasma membrane. PNUT acts as a negative regulator of dPIP5K^L^ activity, thus regulating the levels of PI(4,5)P_2_ present at the microvillar PM. This illustration was created with http://BioRender.com

Given that the enhanced rate of PI(4,5)P_2_ resynthesis on depletion of PNUT (*pnut*^*XP*^*/+*) is suppressed on depletion of dPIP5K^L^, it seems likely that PNUT is a negative regulator of dPIP5K^L^. In principle PNUT could negatively regulate dPIP5K^L^ in distinct ways: (i) PNUT might directly inhibit enzyme activity of dPIP5K^L^; and (ii) depletion of PNUT might improve the accessibility of the substrate PI4P to dPIP5K^L^. We found that the in vitro activity of dPIP5K^L^ in the test tube was enhanced when provided with exogenous lipid substrate and a cell lysate depleted of PNUT and containing dPIP5K^L^. Because the activity on exogenously added PI4P was enhanced in PNUT-depleted lysates, it is possible that PNUT regulates dPIP5K^L^ activity directly. However, it cannot be ruled out that in vivo, loss of PNUT may also improve the accessibility of dPIP5K^L^ to PI4P. Previous studies have presented experimental evidence for septin 2 (Hu et al, 2010) and septin 7 (Ewers et al, 2014) in limiting protein diffusion, but a role for septins in modulating PI(4,5)P_2_ diffusion is unclear (Lee et al, 2014). Nevertheless, our findings strongly support a role for PNUT as a regulator of lipid kinase activity and PI(4,5)P_2_ resynthesis during PLC signalling in vivo (Fig 6). Although we have presented evidence for a role of PNUT (Septin 7) in regulating PI(4,5)P_2_ resynthesis, it is possible that other septins could also regulate this process; genetic tools for more effective depletion of these will aid such studies. Last, it is to be noted that several others proteins that bind PI(4,5)P_2_ are present at the plasma membrane; these include many proteins that regulate the actin cytoskeleton such as profilin, gelsolin, ezrin, and anillin. These could also contribute to PI(4,5)P_2_ resynthesis and their role remains to be investigated.

## Materials and Methods

### Fly stocks

Fly stocks were maintained in 25°C laboratory incubators with 50% relative humidity and no internal illumination. All flies were raised on standard corn meal media containing 1.5% yeast. For all the constant light experiments, flies were grown in an incubator with constant illumination from a white light source for the required time periods.

The wild type used was *Red Oregon-R* (*ROR*). Gal4 UAS system was used for targeted expression of transgenic constructs. The following fly alleles and insertions obtained for the experiments are described here: *so*^*D*^ (#4287; Bloomington Stock), *dPIP5K*^*GSV*^ overexpression line GS200386, with 1X UAS (DGRC-Kyoto), *dPIP5K*^*18*^ (Chakrabarti et al, 2015), *dPIP5K*^*L*^, *dPIP5K*^*L-K189A*^, *dPIP5K*^*L-RNAi*^, *dPIP5K*^*S*^*::HA* and *dPIP5K*^*L*^*::GFP* (generated during this study), *pnut*^*XP*^*/*+, *UAS-Ac*_*Gq*_ (Gaiti Hasan, NCBS), *PNUT*^*RNAi*^ (VDRC: v11791). For the generation of *dPIP5K*^*Bac*^ fly, a bacterial artificial chromosome (BAC) clone encompassing *dPIP5K*, CH321-03B05 (in *attB-Pacman-CmR* vector) was obtained from p[acman] resource (Venken et al, 2006). This BAC clone was 57.178 Kb long and included the *dPIP5K* gene with extended 5′ and 3′ regions having the promoter and the regulatory regions of the gene present in 5′ UTR. The presence of *dPIP5K* in the clone was verified by PCR and this clone containing plasmid was isolated. The isolated plasmid after PCR verification was microinjected into embryos and inserted on third chromosome via ΦC31 integration into the VK00033 attP docking site (BDSC #32543) to generate the *dPIP5K* genomic transgene.

### RNA extraction and Q-PCR

RNA extraction was done from 1-d-old *Drosophila* retinae or heads using TRIzol reagent (Invitrogen). After this, purified RNA was treated with amplification grade DNase I (Invitrogen). Reverse transcription was done using SuperScript II RNase H–Reverse Transcriptase (Invitrogen) and random hexamers (Applied Biosystems). Quantitative PCR (Q-PCR) was performed with the Applied Biosystems 7500 Fast Real-Time PCR instrument. Primers were designed at the exon-exon junction following the parameters recommended for Q-PCR. Transcript levels of the ribosomal protein 49 (RP49) were used for normalization across samples. Three separate samples were collected from each genotype, and duplicate measures of each sample were conducted to ensure the consistency of the data. The following primer pairs were used:

RP49 fwd: CGGATCGATATGCTAAGCTGT
RP49 rev: GCGCTTGTTCGATCCGTA
Sktl fwd: CTCATGTCCATGTGTGCGTC
Sktl rev: TTAATGGTGCTCATCAGTG
dPIP5K fwd: AGCAGAGAAAACCGCTTAGG
dPIP5K rev: GGCGATTCACTGACTTATTCC
dPIP5K^L^ fwd: AGAACCGCACACCACTAATCCC
dPIP5K^L^ rev: GCGGTGCATATGGACCATATGG
dPIP5K^S^ fwd: GGACATTGTGAGCGTTTCGG
dPIP5K^S^ rev: TCTGTCCATGTGGGAGTGGA

### Cloning of *dPIP5K*^*L*^

Total RNA was isolated from fly retinae using the Trizol reagent, following which the RNA was treated with DNAse I amplification grade for digestion of genomic DNA. Full length cDNA clones were obtained using primer specific to the 3′ end of *dPIP5K*^*L*^ and then cloned into an intermediate vector: pCR-XL-TOPO (TOPO XL PCR Cloning Kit; Invitrogen) and finally subcloned into fly expression vector pUAST-attB using Not1 and Xho1 restriction sites. Transgenic flies were obtained upon microinjection. dPIP5K^L-K189A^ (kinase dead) was obtained by doing a site directed mutagenesis at Lysine (K)189 position in dPIP5K^L^. The following primer pairs were used:

To obtain *dPIP5K*^*L*^ cDNA: TGCCTAGCAATACAATGAGC
To clone *dPIP5K*^*L*^ into pUAST-attB:
UAS dPIP5K^L^ Not1_ fwd: GCGGCCGCATGGCCTCCGGCGAT
UAS dPIP5K^L^ Xho1_rev: GAGAGACTCGAGCTAATCCCGTCCCTGTCC
To generate *dPIP5K*^*L*-*K189A*^ kinase dead mutant:
dPIP5K^L-K189A^ fwd: ACGAGTTCATCATAGCGACGGTGCAACACAA
dPIP5K^L-K189A^ rev: ACGAGTTCATCATAGCGACGGTGCAACACAA

### Generation of *dPIP5K*^*L-RNAi*^ flies

The Transgenic RNAi Project (TRiP) guidelines were used to synthesize shRNA constructs against dPIP5K^L^, which were then cloned into Walium 20 vector. Transgenic flies were obtained upon microinjection of the constructs. The following primer pair was used:

dPIP5K^L-RNAi^ fwd: CTAGCAGTGCGTCCTAGAAGACCTCTACCTAGTTATATTCAAGCATAGGTAGAGGTCTTCTAGGACGCGCG
dPIP5K^L-RNAi^ rev: AATTCGCGCGTCCTAGAAGACCTCTACCTATGCTTGAATATAACTAGGTAGAGGTCTTCTAGGACGCACTG

### Electroretinogram (ERG)

Flies were briefly put on ice to anaesthetize and immobilized at the end of a disposable pipette tip such that the head protruded out. Recordings were done using glass microelectrodes filled with 0.8% wt/vol NaCl solution. Voltage changes were recorded between electrode placed at the surface of the eye and the ground electrode placed on the thorax. Before recording, flies were dark adapted for 5 min. Recordings were done with 2-s flashes of green light stimulus, with 10 stimuli (flashes) per recording and 15 s of recovery time between two flashes of light. Green light stimulus was delivered from a LED light source within 5 mm of the fly’s eye through a fibre optic guide. Calibrated neutral density filters were used to vary the intensity of the light source during intensity response experiments. Voltage changes were amplified using a DAM50 amplifier (SYS-DAM50; WPI) and recorded using pCLAMP 10.7 (Molecular Devices). Analysis of traces was performed using Clampfit 10.7. Analysed results were plotted using GraphPad Prism software.

### Pseudopupil assay

To monitor PI(4,5)P_2_ dynamics in live flies, transgenic flies expressing PH-PLCδ::GFP [PI(4,5)P_2_ biosensor] were anaesthetized and immobilized at the end of a pipette tip using a drop of colorless nail varnish and fixed by clay on the stage of an Olympus IX71 microscope. The fluorescent deep pseudopupil (DPP, a virtual image that sums rhabdomere fluorescence from ∼20 to 40 adjacent ommatidia) (Franceschini et al, 1981; Chakrabarti et al, 2015) was focused and imaged using a 10× objective. Time-lapse images were taken by exciting GFP using a 90 ms flash of blue light and collecting emitted fluorescence. The program used for this purpose was created in Micromanager. Following preparation, flies were adapted in red light for 6 min after which the eye was stimulated with a 90 ms flash of blue light (ƛ_max_488 nm). The blue light used to excite GFP was also the stimulus to rapidly convert most of the rhodopsin (R) to metarhodopsin (M), thus activating the phototransduction cascade and triggering depletion of rhabdomeric PI(4,5)P_2_. Between the blue light stimulations, photoreceptors were exposed to long wavelength (red − ƛ_max_660 nm) light that reconverts M to R. The recovery in DPP fluorescence intensity with time indicates translocation of the probe from cytoplasm to rhabdomere membrane upon PI(4,5)P_2_ resynthesis. The DPP intensity was measured using ImageJ from NIH. Cross-sectional areas of rhabdomeres of R1-R6 photoreceptors were measured and the mean intensity values per unit area were calculated. For quantification of recovery, the fluorescence value of the second image was subtracted from all images and this value for the first image was set as 100. The fluorescence value of subsequent images (i.e., n-second) were normalized to the value for first image.

### Optical neutralization

To study retinal degeneration, flies of the desired age and reared under the required experimental conditions were decapitated and the heads fixed on a glass slide using a drop of colorless nail polish. Imaging of the photoreceptors was done using a 40× oil immersion objective lens (BX43; Olympus). Images were acquired by using CellSens software. For light adaptation, newly eclosed flies were transferred to incubators programmed with constant light settings.

### Scoring retinal degeneration

To calculate a quantitative index of degeneration, 50 ommatidia from five different flies of each genotype were counted for each time point. The central photoreceptor, which is UV sensitive, hence did not show any light dependent retinal degeneration, was used as the reference and the rest of the photoreceptors were scored. Each intact rhabdomere was assigned a score of 1, and each degenerated rhabdomere was counted as 0. Hence, the maximum number is 7 and minimum is 1 (representing R7, which is insensitive to white light) Thus, control photoreceptors will have a score of 7 and the genotypes that undergo degeneration will have a score from 1 to 7. Analysed results were plotted using GraphPad Prism software.

### Western blotting

Heads or retinae from 1-d-old flies were harvested and crushed in 2× SDS–PAGE sample buffer followed by boiling at 95°C for 5 min. For protein sample preparation from S2R+ cells, 48 h post transfection, cells were harvested, washed twice with PBS and lysed in 2× SDS–PAGE sample buffer. The genomic DNA was sheared using an insulin syringe followed by boiling the samples at 95°C for 5 min.

Protein extracts were separated using SDS–PAGE and electro blotted onto nitrocellulose filter membrane (Hybond-C Extra; [GE Healthcare]), using semidry transfer apparatus (Bio-Rad). Next, the membrane was blocked in 5% Blotto (sc-2325; Santa Cruz Biotechnology) in PBS with 0.1% Tween 20 (Sigma-Aldrich) (PBST) for 2 h at room temperature. Primary antibody incubation was done overnight at 4°C using the appropriate antibody dilutions. The following antibodies were used: anti-α-tubulin (1:4,000, DHSB [E7c]), anti dPIP5K (1:1,000, lab generated), anti GFP (1:2,000, SC [B2]), anti HA (1:1,000, CST [6E2]), and anti-PNUT (1:250, DSHB [4C9H4-c]). Following this, the membrane was washed thrice in PBST for 10 min each and incubated with 1:10,000 dilutions of appropriate secondary antibody coupled to horseradish peroxidase (Jackson ImmunoResearch Laboratories) at room temperature for 2 h. Next, the membrane was washed thrice in PBST for 10 min each, developed with ECL reagent (GE Healthcare) and imaged in LAS 400 instrument (GE Healthcare).

### Immunohistochemistry

For Immunohistochemistry, retinae from 1-d old flies were dissected in chilled PBS, followed by fixation in 4% paraformaldehyde in PBS with 1 mg/ml saponin at room temperature for 30 min on orbital shaker. Fixed retinae were washed three times in PBTx (1× PBS + 0.3% Triton X-100) for 10 min each. The sample was then blocked using 5% FBS in PBTx for 2 h at room temperature on shaker, after which the sample was incubated with primary antibody in blocking solution overnight at 4°C on a shaker. The following antibodies were used: anti-GFP (1:5,000; Abcam [ab13970]) and anti-HA (1:50, CST [6E2]). Appropriate secondary antibodies conjugated with a fluorophore were used at 1:300 dilutions (Alexa Fluor 488/568/633 IgG; [Molecular Probes]) and incubated for 4 h at room temperature. Wherever required, during the incubation with secondary antibody, Alexa Fluor 568-phalloidin (1:200; Invitrogen [A12380]) was also added to the tissues to stain the F-actin. After three washes in PBTx, samples were mounted in 70% glycerol in 1× PBS. Whole mounted preparations were imaged on Olympus FV3000 confocal microscope using Plan-Apochromat 60×, NA 1.4 objective (Olympus). Image analysis was performed using ImageJ from NIH.

### Cell culture, transfections and dsRNA treatment

*Drosophila* S2R+ cells stably expressing ActGal4 were maintained on Schneider’s media supplemented with 10% non heat inactivated FBS and contained antibiotics-streptomycin and penicillin (Schneider’s Complete Media, SCM). Cell transfections were carried out using Effectene (301425; QIAGEN) as per the manufacturer’s protocol. For *pnut* knockdown experiments, 0.5 million cells were plated in five biological replicates each and allowed to settle overnight. Post this, SCM was removed and 300 μl of Schneider’s incomplete media was added to the cells, followed by addition of 1.875 μg of the desired dsRNA and incubated for 1 h. dsRNA against GFP was used as a control. Post an hour, 300 μl of SCM was added. This procedure was repeated post 48 h, followed by transfections of the cells with *dPIP5K*^*L*^ plasmid, as described above. All dsRNA were synthesized using the MEGAscript RNAi kit (AM1626; Thermo Fisher Scientific). Primers for dsRNA amplification were synthesized in accordance with the DRSC/TRiP Functional Genomics Resources.

The following primer pairs were used:

dsGFP fwd: TAATACGACTCACTATAGGGATGGTGAGCAAGGGCGAGGAG
dsGFP rev: TAATACGACTCACTATAGGGCTTGTACAGCTCGTCCATGCCG
dsPnut fwd: TAATACGACTCACTATAGGGGAAGGAGTGGGAGGATGTCA
dsPnut rev: TAATACGACTCACTATAGGGTTAGGATCAATTTCGCCTCG

### Lipid kinase assay

#### Cell lysate preparation

S2R+ cells expressing the desired constructs were dislodged from plates, harvested by centrifugation at 1,675*g* for 5 min in a table top centrifuge. Then they were washed twice with PBS, and lysed with lysis buffer (50 mM Tris-Cl, 1 mM EGTA, 1 mM EDTA, 1% Triton X-100, 50 mM NaF, 0.27M sucrose, and 0.1% 2-mercaptoethanol) with freshly added protease inhibitor and phosphatase inhibitor cocktail (Roche). Protein equivalent to 10 μg was estimated by Bradford assay.

#### Micelle formation and kinase assay

600 pmoles of 37:4 PI4P (LM1901; Avanti) was dried in LoBind 1.5 ml Eppendorf tubes along with 20 μl 0.5 mM Phosphatidylserine (Sigma-Aldrich) and 200 μl of CHCL_3_ in vacuum for 20 min at 949*g*. After drying of lipids, 50 μl of 10 mM Tris–HCl (pH 7.4) was added to each tube and sonicated at room temperature for 3 min. Next, 50 μl of 2× kinase assay buffer containing 40 μM ATP (Roche) was added to the tubes and 10 μg equivalent of corresponding lysate was added. Immediately after this, the tubes were transferred to a shaking incubator at 30°C for 30 min for the kinase reaction to happen. The reaction was stopped by adding 125 μl of 2.4N HCl to the reaction tubes. Then 250 μl each of CHCl_3_ and methanol were added. After this, the tubes were vortexed for 2 min and centrifuged at 1,000*g* for 5 min to allow a clean phase separation to happen. The upper phase was punctured and the lower organic phase was transferred to a fresh LoBind 1.5 ml Eppendorf. To this tube, 500 μl of Lower Phase Wash Solution (LPWS: methanol/1 M hydrochloric acid/chloroform in a ratio of 235/245/15 [vol/vol/vol]) was added. It was vortexed for 2 min and clean phase separation was obtained by spinning tubes at 1,000*g* for 5 min. Again, the lower organic phase was transferred to a fresh LoBind 1.5-ml Eppendorf tube.

#### Derivatization and LC MS/MS

The organic phase obtained after lipid extraction was directly subjected to derivatization using 2M TMS-diazomethane (Acros). 50 μl TMS-diazomethane was added to each tube and vortexed gently for 10 min at 670*g*. The reaction was neutralized by 10 μl of glacial acetic acid. The samples were dried, and reconstituted in 200 μl of methanol. LC MS/MS run method was used as described in Ghosh et al (2019). The MRM transitions for 37:4 PI4P used was 995.5/613.5 and for 37:4 PI(4,5)P_2_ was 1,103.5/613.5. The area under the peaks for individual lipid species was extracted using MultiQuant software. Numerical analysis was performed in Microsoft Excel and the graphs were plotted using GraphPad Prism software.

### Immunofluorescence from S2R+ cells

48 h post transfection with *dPIP5K*^*L*^*::GFP*, cells were fixed in 2.5% PFA for 20 min. Upon fixation, cells were washed thrice with PBTx (1× PBS + 0.3% Triton X-100) for 10 min each, permeabilized with 0.37% Igepal (Sigma-Aldrich) in 1× M1 for 13 min and then blocked for 1 h at room temperature in 1× M1 containing 5% FBS and 2 mg/ml BSA. Post blocking, the cells were incubated with primary antibodies in blocking solution overnight at 4°C. The following antibodies were used: anti-GFP (1:5,000; Abcam [ab13970]) and anti-PNUT (1:250; DSHB [4C9H4]). Next morning, the cells were washed thoroughly, and incubated with the appropriate secondary antibodies conjugated with fluorophore at 1:300 dilutions (Alexa Fluor 488/568/633 IgG, [Molecular Probes]) and incubated at room temperature for 2 h. The cells were then washed and imaged on an Olympus FV3000 confocal microscope using Plan-Apochromat 60×, NA 1.4 objective (Olympus). Image analysis was performed using ImageJ from NIH.

### Lipid-binding assay

48 h post transfection, S2R+ cells expressing UAS-PNUT were harvested and washed twice with PBS. Then the cells were sheared in fat blot buffer (50 mM Tris–HCl, pH 7.5, 150 mM NaCl) using an insulin syringe. Strips of nitrocellulose membranes (Hybond-C Extra; [GE Healthcare]) were spotted with increasing picomoles of PI4P and PI(4,5)P_2_ (Echelon lipids [P-4016 and P-4516]). The spotted membranes were dried for 1 h and blocked using 5% blotto (HiMedia) in fat blot buffer for 1 h 30 min at room temperature. Then, the strips were incubated overnight at 4°C with the cell lysate. Next morning, the membranes were washed extensively five times with fat blot buffer with 0.1% tween and then incubated with anti PNUT (1:250; DSHB [4C9H4]) for 3 h at room temperature. The membranes were then probed with HRP-conjugated anti mouse secondary antibody (1:10,000; Jackson Immunochemicals). Binding was detected using ECL (GE Healthcare) on a LAS4000 instrument.

### Statistical analysis

All statistical analyses were performed in GraphPad Prism 10 software. Unpaired two-tailed *t* test with Welch correction, one-way ANOVA, followed by Tukey’s multiple-comparisons test or two-way ANOVA grouped analysis with Bonferroni’s post multiple comparisons were carried out where applicable.

## Supplementary Material

Reviewer comments
